# Assessment of Dentin Hypersensitivity in Patients With Bulimia Nervosa

**DOI:** 10.7759/cureus.80449

**Published:** 2025-03-12

**Authors:** Mariyana Kirova, Tsvetelina Borisova-Papancheva

**Affiliations:** 1 Department of Conservative Dentistry and Oral Pathology, Medical University of Varna, Varna, BGR

**Keywords:** bulimia nervosa, dental erosion, dentin hypersensitivity, self-induced vomiting, visual analogue scale (vas)

## Abstract

Summary: The article presents results obtained from a study on dentin hypersensitivity in patients diagnosed with bulimia nervosa. These patients are characterized by erosions of the hard dental tissues caused by frequent acid exposure due to self-induced vomiting. Erosive lesions are often associated with hypersensitivity due to dentin exposure.

Objective: The aim of the study is to determine the presence of a direct relationship between dentin hypersensitivity and dental erosion in patients with bulimia nervosa, as erosion plays an important role in the localization of dentin hypersensitivity.

Materials and methods: The study included 30 patients diagnosed with bulimia. We examined 788 teeth, of which erosions were registered in 316. Two methods were used to provoke dentin hypersensitivity: a tactile stimulus and a cold stimulus. Pain assessment was performed using the Visual Analogue Scale (VAS). The study was conducted at the Dental Center of the Faculty of Dental Medicine in Varna, as well as in the Clinical Halls of 'Conservative Dentistry and Oral Pathology' within the Faculty of Dental Medicine, during the period 2023-2024.

Results: A significant proportion of the examined patients (96.67%) reported experiencing dentin hypersensitivity in at least one tooth. Patients most commonly reported pain triggered by thermal stimuli, particularly cold, as well as during tooth brushing. After examining all available teeth with erosion, we found that nearly half of them (47.15%) were hypersensitive.

Conclusion: Dental erosion in patients with bulimia nervosa plays a crucial role in the localization of hypersensitivity. The severity of hypersensitivity may vary, ranging from mild discomfort to intense pain, which can impair masticatory and speech functions, as well as interfere with the patient’s daily activities.

## Introduction

Dentin hypersensitivity is the second most common oral symptom in patients with bulimia, following dental erosion, which plays a significant role in the localization of hypersensitivity. One of the primary contributing factors is frequent self-induced vomiting, which leads to demineralization and the gradual destruction of enamel, resulting in dentin exposure [1-6]. The highly acidic pH of gastric acid contributes to the degradation of the so-called smear layer, a protective layer that plays a crucial role in dentin protection [7,8]. This layer is a thin organic film that forms an adhesive matrix on mineralized dental tissues. The salivary pellicle acts as a barrier against external stimuli and protects the dentin tubules from direct exposure to aggressive factors [9]. However, frequent acid exposure leads to its rapid degradation, increasing the risk of hypersensitivity [10,11].

Dentin hypersensitivity is characterized as a transient, sharp pain arising from exposed dentin in response to thermal, tactile, evaporative, osmotic, or chemical stimuli, which cannot be attributed to any other dental pathology or structural defect [12,13]. A characteristic feature of dental hypersensitivity is an excessive response to sensory stimuli that typically do not provoke a reaction in healthy teeth [14]. Dental hypersensitivity can occur across all age groups but is more commonly observed in young and middle-aged individuals [15]. According to some studies, the most affected age group is between 25 and 29 years [16].

Endogenous acids, which are a primary etiological factor in patients with bulimia, act as erosive agents contributing to the development and progression of hypersensitivity. Their effect can lead to the exposure of dentinal tubules, further increasing tooth sensitivity [17].

The study of tooth hypersensitivity has been the subject of research for many years. Some studies have identified differences between teeth with hypersensitivity and those without. In teeth without hypersensitivity, very few exposed dentinal tubules are observed, whereas in hypersensitive teeth, the number of dentinal tubules per unit area is significantly higher. On root surfaces, the number of dentinal tubules has been found to be up to eight times greater [18].

Establishing an accurate diagnosis of dentin hypersensitivity requires a significant amount of time, as it involves a detailed dental history and relies on the process of eliminating other similar conditions through differential diagnosis [19,20]. For a more precise diagnosis, additional information is gathered regarding the characteristics of pain, including its localization, nature, intensity, duration, and the type of stimulus that triggers it. The most common pain triggers include exposure to cold and acidic foods and beverages, as well as contact with cold air [21].

The clinical examination includes the identification of exposed dentin through visual-tactile assessment of the teeth, as well as the application of various stimuli to provoke hypersensitivity. Among the available methods for assessing dentin hypersensitivity, air blast tests and tactile tests are most commonly preferred due to their physiological basis and reproducibility [22,23].

## Materials and methods

In order to fulfill the research objective, a study was conducted on 30 patients with a confirmed diagnosis of bulimia nervosa who required dental treatment. The Committee on Research Ethics issued approval 140/01.02.2024. The selected patients did not have any severe concomitant systemic diseases.

The exclusion criteria for this study included pregnancy, individuals under the legal age of adulthood, patients who had undergone prior treatment for hypersensitivity within the past three months, as well as those who had taken analgesics, antihistamines, anticonvulsants, sedative medications, or had been undergoing anti-inflammatory therapy within the last 72 hours. Additionally, teeth presenting with carious lesions, pulpitis, devitalization, fractures, congenital defects, or crowns were excluded from the study.

Prior to the intraoral examination, each patient underwent a structured survey to assess the presence of dentin hypersensitivity and the factors triggering it. Additionally, all patients received professional oral hygiene, including the removal of dental calculus and plaque.

To induce and evaluate dentin hypersensitivity during the study, two standardized methods were employed: tactile stimulation and cold stimulation.

Tactile stimulation is performed by gently moving the tip of a periodontal probe in a linear motion from the mesial to the distal direction in the cervical area of the buccal surface of the tooth, applying moderate pressure. The same method is then used to examine the occlusal surface of distal teeth, as well as the palatal and lingual surfaces of both maxillary and mandibular teeth, ensuring a comprehensive assessment of all tooth surfaces.

The cold stimulus is applied by directing an air jet from the dental unit perpendicularly onto the cervical area of the tooth from a distance of 3-4 mm for a duration of three seconds. Subsequently, the air jet is redirected at a 90-degree angle to the occlusal or incisal surface of the tooth for the same duration. Finally, the procedure is repeated on the palatal and lingual surfaces to ensure comprehensive assessment of all tooth surfaces.

To minimize the risk of false results during the assessment of a tooth with reported hypersensitivity, neighboring teeth were isolated using cotton rolls, Teflon tape, or a gingival barrier.

Each stimulus was applied only once per tooth. The applications were performed at one-minute intervals.

After each stimulus, participants were instructed to report the intensity of pain, using the following assessment scale: the Visual Analogue Scale (VAS). The VAS is a method for subjective pain assessment that allows patients to determine the intensity of their pain independently. The scale is a linear graphical system where patients mark their pain level along a continuum from 'no pain' to 'maximum pain.' The scale is typically graduated in millimeters (0-100 mm) or centimeters (0-10 cm).

The patient selects a point from 0 to 100 mm based on their pain perception, ranging from 'no pain' to 'maximum pain.' The interpretation of the scale is as follows: No pain: 0 cm; Mild pain: 1 to 3 cm; Moderate pain: 4 to 6 cm; Severe pain: 7 to 9 cm; Extremely severe pain: 10 cm.

The data from the study were entered and analyzed using the advanced functions of the mathematical-statistical software package SPSS v.22 (IBM Corp., Armonk, NY, USA) and Microsoft Excel (Redmond, WA, USA), utilizing the Data Analysis tool.

## Results

The total number of teeth examined in the patients participating in our study was 788. However, after excluding 153 teeth that did not meet the inclusion criteria - namely, carious, pulpitic, devitalized, fractured teeth, as well as teeth with congenital defects or crowns - a total of 631 teeth were analyzed.

Among these, erosions were identified in 316 teeth, while hypersensitivity was reported in 149 teeth. Almost all of the examined patients reported hypersensitivity in at least one tooth.

**Figure 1:**
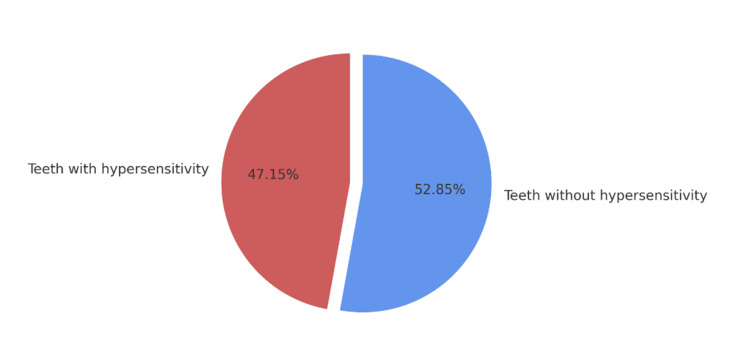
Percentage distribution of teeth with and without hypersensitivity

After examining all available teeth, with a more detailed focus on those with erosion, the collected data were entered into the SPSS data analysis software and Microsoft Excel. The study found that almost half of the teeth with erosive defects showed hypersensitivity. The diagram presented in Figure 1 is based on the data collected through the clinical examination, which assessed the presence of hypersensitivity in teeth with identified erosive defects.

A very high prevalence of hypersensitivity was observed. It was present in 47.15% of teeth with erosive defects, while 52.85% did not exhibit hypersensitivity.

**Figure 2:**
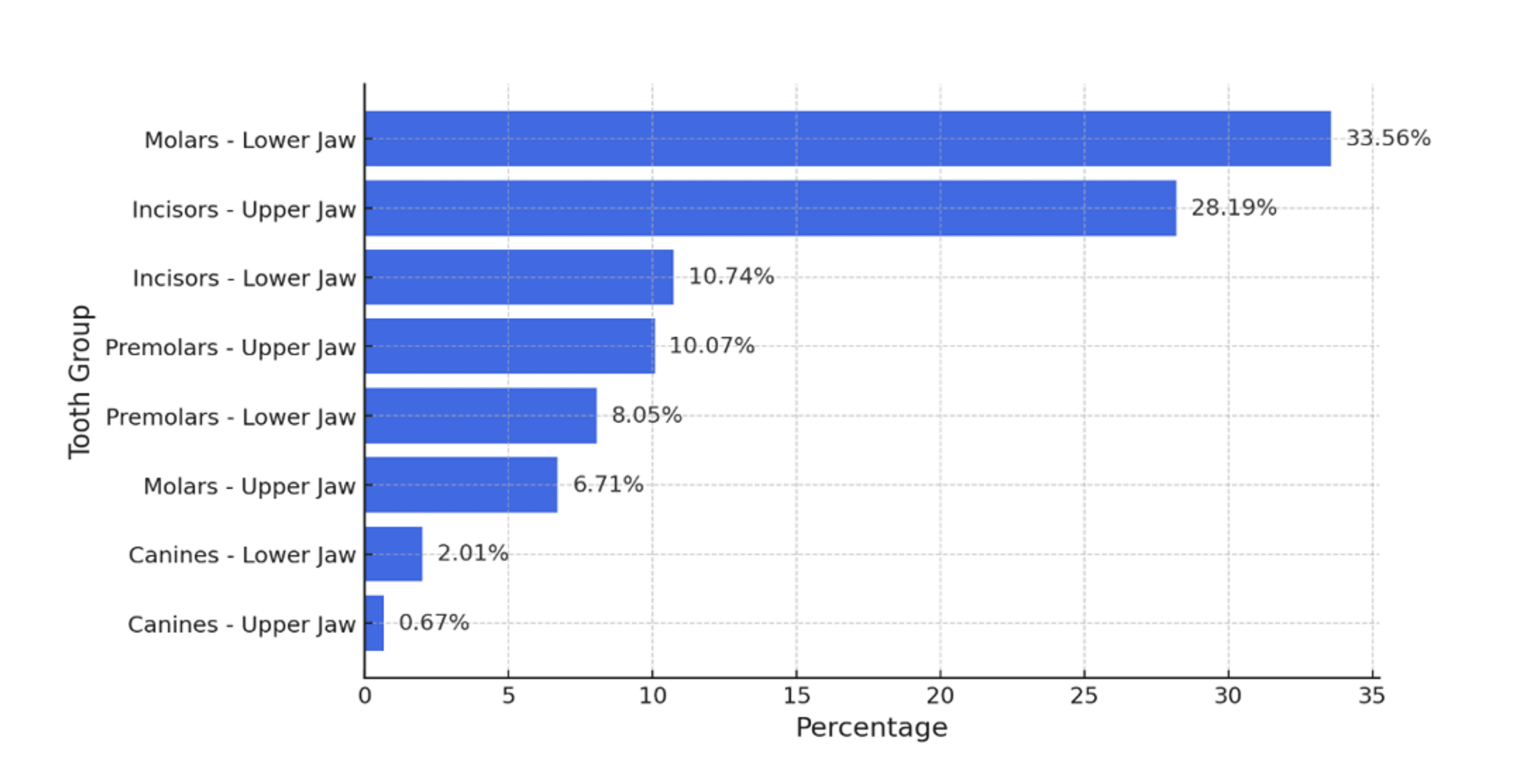
Percentage distribution of hypersensitivity across tooth groups in the upper and lower jaw

The data collected from the examination of all tooth groups in the maxilla and mandible were entered into Microsoft Excel. A horizontal bar chart was generated to provide a clear representation of the percentage distribution of teeth exhibiting dentin hypersensitivity across different tooth groups, stratified by upper and lower jaw (Figure 2).

The data presented in Figure 2 indicate that the molars of the lower jaw are the most affected tooth group in terms of hypersensitivity, exhibiting the highest percentage of affected teeth, 33.56%. The second most frequently affected group is the maxillary incisors, with 28.19% of these teeth demonstrating hypersensitivity. The mandibular incisors and maxillary premolars exhibit similar levels of hypersensitivity, approximately 10%. In contrast, the mandibular premolars show a lower prevalence, with 8.05%, placing them among the less affected tooth groups. Notably, there is a distinct contrast between the molars of the upper and lower jaw - while the mandibular molars are the most severely affected, the maxillary molars demonstrate a significantly lower prevalence of hypersensitivity, recorded in only 6.71% of cases. The canine teeth group is the least affected by hypersensitivity. In the lower jaw, a prevalence of 2.01% was observed, while in the upper jaw, the percentage was 0.67%, indicating an almost negligible presence of hypersensitivity within this tooth group.

**Figure 3:**
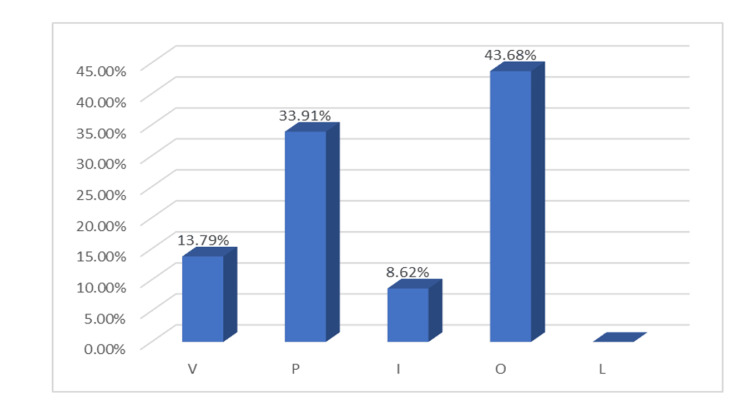
Percentage distribution of hypersensitivity across tooth surfaces V- Vestibular; P- Palatal; I- Incisal; O- Occlusal; L- Lingual surfaces

For a more detailed analysis, data on tooth surfaces were also included. The vestibular and palatal surfaces of the maxillary teeth, as well as the incisal, occlusal, and lingual surfaces of the mandibular teeth, were examined (Figure 3).

The occlusal surfaces of the posterior teeth exhibited the highest prevalence of dentin hypersensitivity, affecting 43.68% of the examined cases. The second most affected surfaces were the palatal surfaces of the maxillary teeth, with a prevalence of 33.91%. Vestibular surfaces were also impacted, with hypersensitivity observed in 13.79% of cases. Incisal surfaces were affected by hypersensitivity primarily when combined with palatal erosion, accounting for 8.62% of cases. No hypersensitivity was observed on the lingual surfaces.

In conducting the study, two methods were employed to induce and subsequently assess dentin hypersensitivity. The first method involved the use of a tactile stimulus, applied with a dental periodontal probe, while the second method utilized a cold stimulus, delivered through a directed air jet from the dental unit. To assess the intensity of the induced pain in our study, we utilized the VAS, a widely employed tool in clinical research.

**Figure 4:**
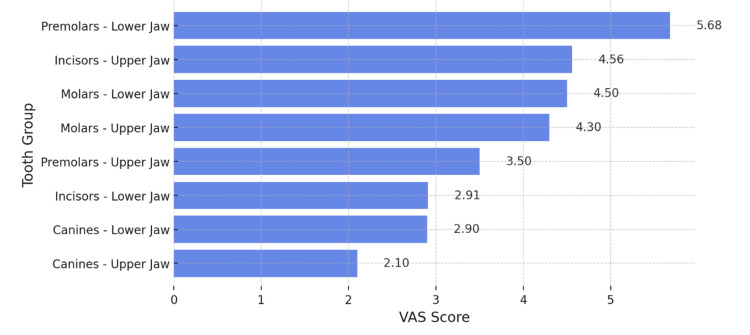
Analysis of Dentin Hypersensitivity Intensity Using the Visual Analogue Scale (VAS), Induced by Tactile Stimulus

The intensity of hypersensitivity was initially assessed in response to the tactile stimulus (Figure 4).

The graph presented in Figure 4 illustrates the mean values of hypersensitivity across different tooth groups, measured using the VAS scale following the application of a tactile stimulus with a probe. The mandibular premolars exhibited the highest recorded pain intensity, with a mean value of 5.68, which is significantly higher compared to the other groups. The maxillary incisors were the second most affected group, with a mean score of 4.56. They were followed by the mandibular and maxillary molars, which demonstrated comparable mean values of 4.5 and 4.3, respectively. The first four groups in the graph correspond to the "moderate pain" category. The mean score recorded for the maxillary premolars was 3.5, notably lower than that observed in the corresponding mandibular premolars. The mandibular and maxillary incisors, as well as the canines, were categorized under the "mild pain" group, with mean values ranging between 2.1 and 2.91.

**Figure 5:**
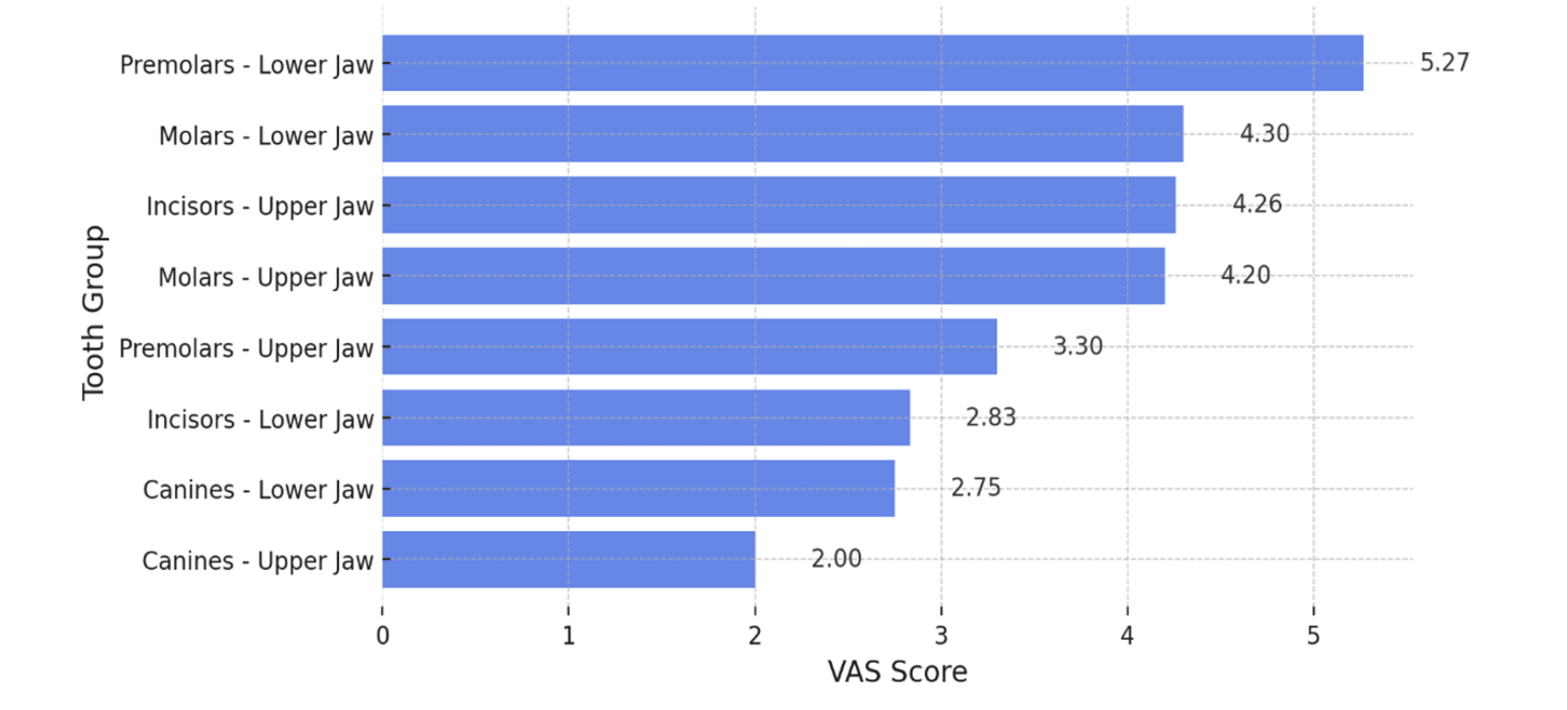
Analysis of Dentin Hypersensitivity Intensity Using the Visual Analogue Scale (VAS), Induced by Cold Stimulus

The following analysis assesses the intensity of hypersensitivity as measured by the application of a cold stimulus (Figure 5).

The results of the study, presented in Figure 5, indicate that the highest VAS scores following cold stimulus application were recorded in the mandibular premolars, with a mean measured value of 5.27. A similar intensity of induced hypersensitivity was observed in the mandibular molars, maxillary incisors, and maxillary molars, with mean values of 4.3, 4.2, and 4.26, respectively. These values fall within the "moderate pain" category. The recorded mean value for the maxillary premolars was 3.3, which is lower compared to the corresponding mandibular premolars, suggesting a less pronounced degree of hypersensitivity. The lowest hypersensitivity values were observed in the mandibular incisors and the canines of both jaws. In cases where hypersensitivity was detected in these tooth groups, it exhibited mild intensity, with recorded values falling within the "mild pain" range, as defined by the VAS scale (1 to 3).

The results obtained after provocation with two types of stimuli are similar, with the VAS scale values measured during the tactile stimulus being slightly higher, however, after stimulation with both stimuli, the investigated hypersensitivity is determined to be "moderate".

## Discussion

In our conducted study, we identified a high prevalence of dentin hypersensitivity among patients suffering from bulimia, with the most affected teeth being those exhibiting enamel and dentin erosions. Dentin hypersensitivity manifests with a single symptom - pain in teeth unaffected by caries. The symptom can range from mild irritation and discomfort to sharp, intense pain. Our observations indicated that the most common triggers of hypersensitivity were cold thermal stimuli and mechanical stimuli, which were clinically replicated through exposure to cold air from the dental unit and scratching the tooth surface with a probe [24]. The pain may persist only while the stimulus is present or may last for a few seconds beyond its removal.

We found that the molar group was the most frequently affected by hypersensitivity, however, the intensity of dentin hypersensitivity was not the highest in these teeth. The highest degree of hypersensitivity was recorded in the mandibular premolars, followed by the incisors of the upper and lower jaw. The results obtained from stimulation with a tactile stimulus were slightly higher than those recorded with a cold stimulus. However, in both cases, the mean values corresponded to "moderate pain" according to the VAS scale. Regarding the surfaces that exhibited the highest sensitivity, we found that the occlusal surfaces of the distal teeth demonstrated the greatest hypersensitivity, followed by the palatal surfaces of the upper anterior teeth.

We established that erosive lesions are a predisposing factor for hypersensitivity due to the demineralization of enamel and dentin [25] and consider the presence of acids to be the primary contributing factor to the development of dental erosions and hypersensitivity, a finding that has also been confirmed in other studies [26].

The average measured results obtained using the widely utilized VAS scale for clinical research purposes were approximately 4, which is consistent with the findings reported by other authors.

Limitations of the study

Sample Size

Although the study includes 30 patients diagnosed with bulimia, which is a relevant number for clinical research in this field, larger sample sizes could further confirm the observed trends and provide greater statistical significance.

Subjective Nature of Pain Assessment

The evaluation of hypersensitivity is based on self-reported pain levels using the VAS scale, which is a subjective method. Individual differences in pain perception and reporting may influence the results.

## Conclusions

The results of the study indicate that dentin hypersensitivity is a common issue among patients suffering from bulimia nervosa, particularly in teeth affected by enamel and dentin erosions. Erosive lesions are a predisposing factor for the development of dentin hypersensitivity, which is closely linked to the acid exposure of the hard dental tissues characteristic of bulimia. The primary triggers of dentin hypersensitivity are cold thermal stimuli and mechanical stimuli.

After analyzing the results of our study, we consider dentin hypersensitivity to be a clinical indicator of an active erosive tooth wear process in patients with bulimia nervosa.
